# Seasonal Response of North Western Pacific Marine Ecosystems to Deposition of Atmospheric Inorganic Nitrogen Compounds from East Asia

**DOI:** 10.1038/s41598-018-27523-w

**Published:** 2018-06-29

**Authors:** Fumikazu Taketani, Maki N. Aita, Kazuyo Yamaji, Takashi Sekiya, Kohei Ikeda, Kosei Sasaoka, Taketo Hashioka, Makio C. Honda, Kazuhiko Matsumoto, Yugo Kanaya

**Affiliations:** 1 Research and Development Center for Global Change, Japan Agency for Marine-Earth Science and Technology (JAMSTEC), 3173-25 Showa-machi, Kanazawa-ku, Yokohama, Kanagawa 236-0001 Japan; 2 Graduate School of Maritime Sciences, Kobe University, 5-1-5 Fukae-minamimachi, Higashinada-ku, Kobe, Hyogo 658-0022 Japan; 3 Project Team for HPC Advanced Predictions utilizing Big Data, Japan Agency for Marine-Earth Science and Technology (JAMSTEC), 3173-25 Showa-machi, Kanazawa-ku, Yokohama, Kanagawa 236-0001 Japan; 4 Center for Global Environmental Research, National Institute for Environmental Studies, 16-2 Onogawa, Tsukuba, Ibaraki 305-8506 Japan

## Abstract

The contribution of the atmospheric deposition of inorganic nitrogen compounds produced in East Asia to the marine ecosystems of the North Western Pacific Ocean (NWPO) was investigated in this study using a 3-D lower trophic-marine ecosystem model (NEMURO) combined with an atmospheric regional chemical transport model (WRF-CMAQ). The monthly mean values for the wet and dry deposition of nitrogen compounds, including gases (HNO_3_ and NH_3_) and aerosol particles (NO_3_^−^ and NH_4_^+^), were determined using the WRF-CMAQ for the NWPO from 2009–2016. These values were input into the NEMURO as an additional nitrogen source. The NEMURO indicated that the annual average chlorophyll mass concentration at the surface in the subtropical region (20°N–30°N; 125°E–150°E) of the NWPO increased from 0.04 to 0.10 mg/m^3^. Similarly, the gross primary productivity, integrated over sea depths of 0–200 m, increased from 85 to 147 mg C/m^2^/day because of this deposition. This study indicates that the supply of atmospheric inorganic nitrogen compounds from East Asia to the NWPO could have a high nutrient impact on the marine ecosystem in the subtropical region.

## Introduction

The wet and dry depositions of airborne natural and anthropogenic material transported from continents to the ocean surface have become important pathways for supplying nutrients to the phytoplankton biomass, along with upwellings and river discharge^1–7^. Thus, ways to estimate both the atmospheric inputs of nitrogen to the oceans and the impact that atmospheric deposition has on ocean biogeochemistry have been investigated in several studies^2,4,6,8–12^. Duce *et al*. suggested that atmospheric nitrogen deposition is important but has strong geographic gradients^2^. For example, their estimate for nitrogen deposition in 2000 was >700 mg/m^2^/year in the downwind region of major cities of Asia, India, and North America^2^. In East Asia, over the last several decades, anthropogenic emissions have dramatically increased because of the acceleration of industrialization^13^. Itahashi *et al*. reported that the emissions of nitrogen oxides (NO_x_), which can produce nitric acid (HNO_3_), aerosol nitrate (NO_3_), and other nitrogen compounds through photochemical reactions, were rapidly increasing in East Asia, especially in China^14^. This finding suggests there is a large input of pollutants from the Asian continent via atmospheric long-range transport to the region of the North Western Pacific Ocean. Numerous studies have measured the aerosol particle and gas concentrations (e.g., N, P, Si, and Fe compounds), using ground- or ship-based observations in the coastal areas of East Asia and the North Western Pacific Ocean to investigate the potential effects that these compounds have on the marine ecosystem^15–25^.

The deposition of nitrogen compounds into the marginal seas of East Asia and the open ocean of the North Western Pacific Ocean has been estimated using regional/global models^26–29^. Recently, Itahashi *et al*. indicated that the combined wet and dry deposition amount of NO_3_^−^ +HNO_3_ was 1407 Gg N/year in the oceanic regions of East Asia^27^. This corresponds to approximately half of the amount of anthropogenic nitrogen compounds emitted from China. The authors also estimated that 252 Gg N/year was delivered as input into the East China Sea^27^. This value corresponds to 59% of the nitrate discharged by the Yangtze River into the East China Sea^27^. Zhang *et al*. also estimated the atmospheric wet and dry deposition of inorganic nitrogen (NO_3_^−^, NH_4_^+^, HNO_3_, NO_x_, and NH_3_) into the East China Sea^29^. They conducted a simple estimation based on new primary productivity related to atmospheric deposition and reported that the levels of ammonium-nitrogen inputs from atmospheric deposition to the East China Sea were almost the same as those from river discharge. The authors concluded that atmospheric nitrogen deposition can increase new biological primary productivity by 1.1–3.9%. Such estimates clearly indicate that atmospheric deposition cannot be neglected as a source. Thus, we speculate that the emissions of nitrogen compounds from East Asia have a high potential to affect the surface marine ecosystem in the open ocean of the North Western Pacific Ocean.

Onitsuka *et al*. studied the influence of atmospheric nitrogen compound inputs on primary productivity in the Sea of Japan, which is a semi-enclosed marginal sea^9^. They used an ecosystem model combined with an atmospheric regional chemical transport model and showed that atmospheric compounds made a large contribution to new productivity in the southern region of the Sea of Japan, especially in locations with nutrient depletion in the surface layer. They also simulated changes in the nitrogen compound deposition rate from the atmosphere, showing that primary productivity increased linearly as this rate increased. However, their marine ecosystem model^30^ consisted of only four components: dissolved inorganic nitrogen (nutrients), phytoplankton, zooplankton, and detritus. This is clearly a simplified system (e.g., nitrogen compounds, such as ammonium and nitrate, are not separated). In low-nitrogenated waters, ammonium may be the dominant nitrogen source used by phytoplankton^31^. Therefore, the availability of ammonium and nitrate as nutrients in the marine ecosystem would depend on the setting^32^. This finding suggests that a more detailed analysis is needed to understand the role that various inorganic nitrogen compounds derived from the atmosphere play in the marine ecosystem.

Because the surface layer of the subtropical region of the North Western Pacific Ocean appears to always be nutrient-depleted^33^, we estimated the influence of atmospheric nutrient input in this region. To investigate the influence of deposition of inorganic nitrogen compounds derived from the East Asian continent on the marine ecosystem in the North Western Pacific Ocean, we performed numerical simulations with and without the atmospheric deposition of inorganic nitrogen compounds using a 3-D low trophic-marine ecosystem model coupled to an atmospheric regional chemical transport model.

## Results and Discussion

### Atmospheric inorganic nitrogen deposition

**Figure 1:**
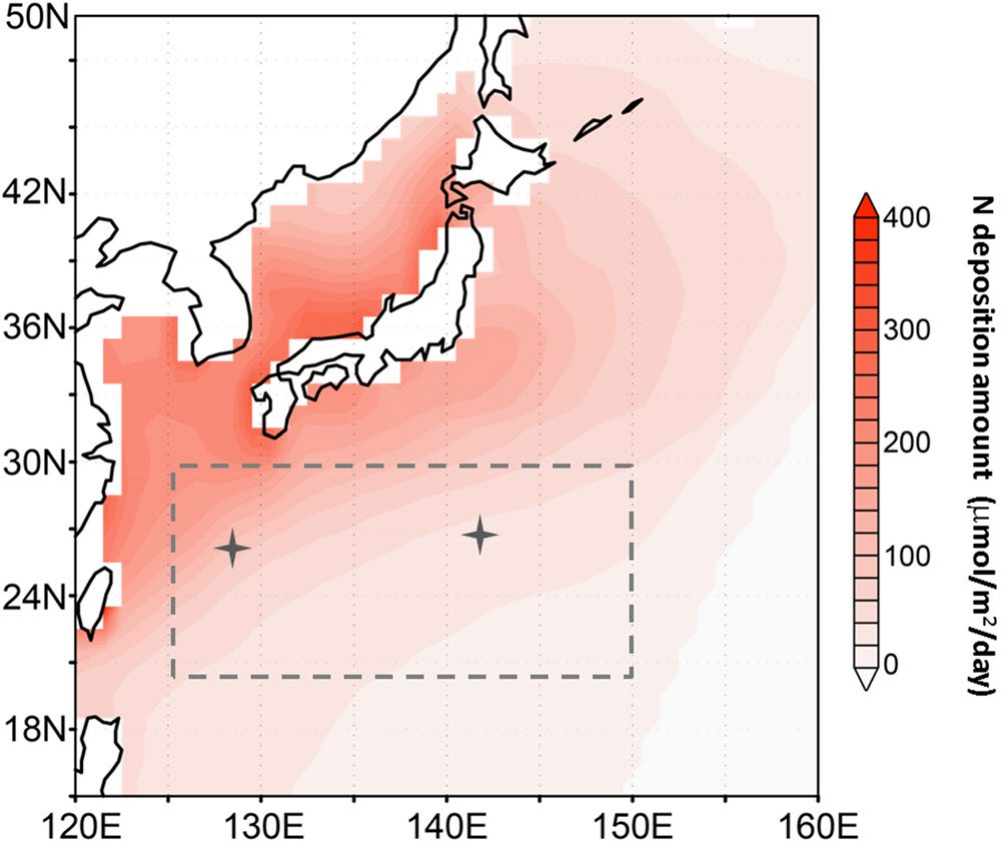
Annual mean spatial deposition of total inorganic nitrogen compounds to the North Western Pacific Ocean, calculated by the WRF-CMAQ model. The focus area (20–30°N, 125–150°E) is demarcated by a dashed gray line. Gray crosses indicate locations of the Acid Deposition Monitoring Network in East Asia (EANET) sites of Ogasawara (27.05°N, 142.13°E) and Hedo (26.52°N, 128.15°E). The figure is created using the Grid Analysis and Display System (GrADS) Version 1.9b4 (http://cola.gmu.edu/grads/).

Figure 1 shows the annual mean spatial distribution of total nitrogen nutrient deposition into the North Western Pacific Ocean in the COCO-NEMURO. This represents the sum of NH_4_ (=NH_4_^+^ +NH_3_) and NO_3_ (=NO_3_^−^ +HNO_3_), averaged for 2009–2016, as calculated by the WRF-CMAQ model. Inorganic nitrogen compound depositions were high in marginal sea areas but decreased with increasing distance from the continent. In this study, we focused on the subtropical area indicated in Fig. 1, covering 20–30°N and 125–150°E. To assess the reproducibility of the WRF-CMAQ model, data from the Acid Deposition Monitoring Network in East Asia (EANET) (http://www.eanet.asia/product/index.html) at Ogasawara (27.05°N, 142.13°E) and Hedo (26.52°N, 128.15°E) were used; they these locations are within our focus area (Fig. 1). We compared the simulated monthly mean wet deposition data (NO_3_^−^ and NH_4_^+^ concentrations in precipitation) extracted from the nearest data point within our model with the EANET data obtained from 2009–2015, as shown in Fig. S1. This comparison indicated that the data in our model captured the seasonality but were slightly lower than EANET values. Generally, our model was in good agreement with the EANET data.

**Figure 2:**
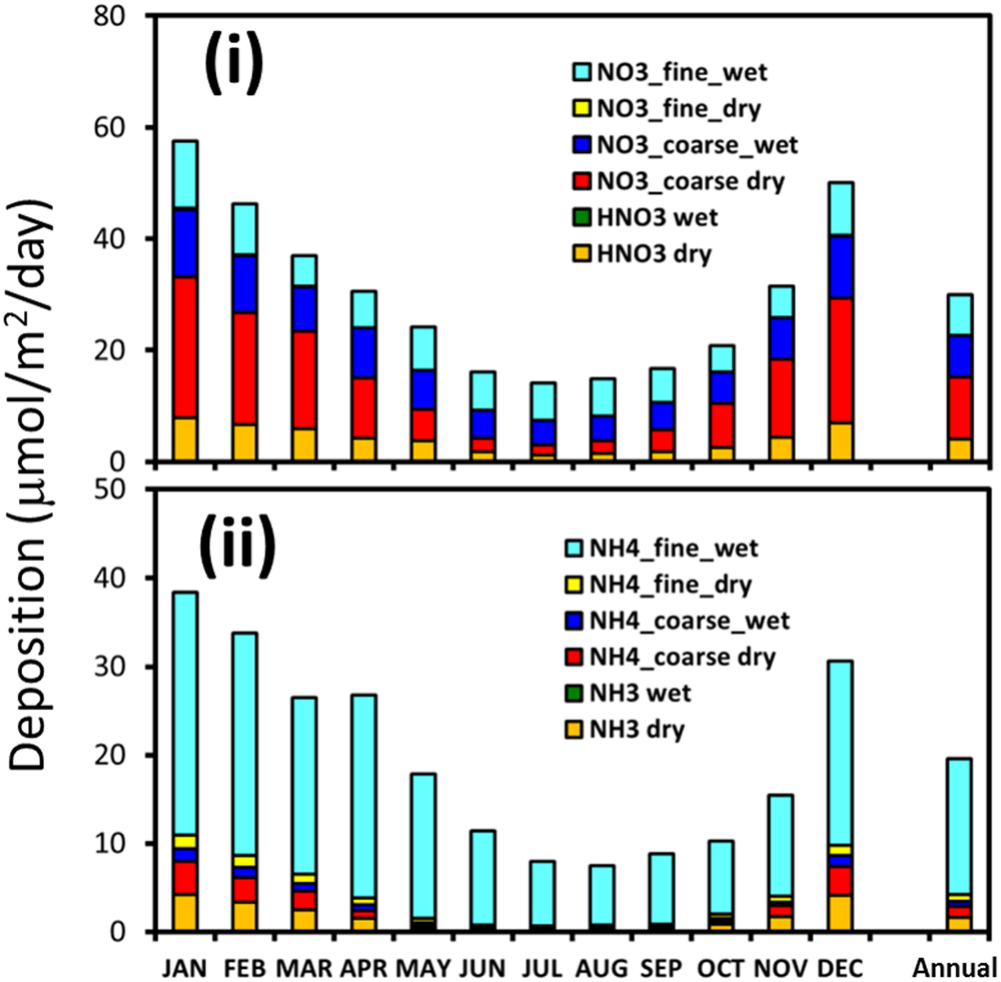
Monthly and annual mean deposition components of the inorganic nitrogen compounds to the focus area (20–30°N, 125–150°E), calculated by the WRF-CMAQ model. Upper and lower panels show the results for (i) NH_4_, and (ii) NO_3_.

The monthly and annual mean deposition amounts are shown in Fig. 2, averaged for 2009–2016 for each inorganic nitrogen compound within the subtropical area. They clearly indicate seasonality and have high and low deposition amounts in winter and summer, respectively. The total depositions of NH_4_ and NO_3_ and the fractions calculated for each depositional process within the subtropical area are listed in Tables S1 and S2. For NH_4_ deposition, the ratio of fine-mode wet deposition to the total NH_4_ was in the range of 68–93%, with an annual average of 79% for this subtropical area. This result suggests that the wet deposition of (NH_4_)_2_SO_4_ is an important source of NH_4_ from the atmosphere^34^. For NO_3_ deposition, both dry and wet coarse-mode depositions were dominant, suggesting the importance of the reaction of nitric acid (HNO_3_) with the sea salt particles. Itahashi *et al*. reported an estimated annual mean NO_3_^−^ deposition to the North Western Pacific Ocean for 2002–2004, which was calculated using the WRF-CMAQ model with almost the same settings as ours^27^. Their total deposition amounts and deposition fractions for a nearby area were almost the same as those found for our study area.

The sources contributing to the focus area were analyzed using an emission sensitivity analysis^35^ of data from 2010. This analysis indicated that the annual mean NH_4_ depositions derived from China, Korea, and Japan were 56%, 5%, and 4%, respectively, and that the NO_3_ depositions were 58%, 5%, and 10%, respectively. This result indicates that the inorganic nitrogen compounds deposited within the focus area were dominantly from East Asia. Additionally, the contributions of NH_4_ and NO_3_ from natural sources (biomass burning and volcanoes) were estimated to be less than 10%, indicating that the deposition of inorganic nitrogen compounds within the focus area mainly reflects anthropogenic sources.

### Surface nitrogen nutrient concentrations

The monthly and annual means for the nitrogen nutrient concentrations at the sea surface (0–9 m) for the subtropical focus area are shown in Fig. S2 and were calculated using the COCO-NEMURO in CTL and N-depo cases, representing simulations without and with the deposition of atmospheric inorganic nitrogen compounds, respectively. The differences and percentage increases in nitrogen nutrient concentrations between the CTL and N-depo cases are also listed in Table 1. The NH_4_ and NO3 concentrations at the sea surface in the N-depo case were still below 0.02 and 0.6 µmol_N/L, respectively. This result suggests that this area may always be depleted of nitrogen nutrients, thus creating a nitrogen-limited state at the sea’s surface in all seasons. The monthly NO_3_ concentrations in the N-depo case were almost the same as those in the CTL case. In contrast, the NH_4_ concentrations were three times higher than those in the CTL case, suggesting that atmospheric deposition can perturb the surface nitrogen nutrient concentrations.

**Table 1:** Summary of the monthly and annual mean differences in each component between cases with and without nitrogen deposition in the subtropical focus area.

Month	Difference^a^ (percentage increase^b^)					
	NH_4_^c,d^	NO_3_^c,d^	PS^c,e^	PL^c,e^	Chl^c,e^	GPP^f,g^
Jan	0.010 (3.1)	0.069 (1.2)	0.043 (3.0)	0.007 (1.8)	0.05 (2.6)	55 (2.7)
Feb	0.010 (3.1)	0.094 (1.2)	0.050 (3.4)	0.007 (1.7)	0.06 (2.8)	69 (2.9)
Mar	0.012 (3.4)	0.069 (1.2)	0.067 (2.9)	0.013 (1.9)	0.08 (2.7)	101 (2.7)
Apr	0.017 (3.3)	0.010 (1.0)	0.054 (1.9)	0.026 (2.5)	0.08 (2.0)	111 (1.9)
May	0.013 (2.6)	0.034 (1.1)	0.030 (1.6)	0.015 (1.7)	0.04 (1.6)	71 (1.5)
Jun	0.010 (2.4)	0.026 (1.1)	0.040 (2.1)	0.016 (2.0)	0.06 (2.1)	60 (1.4)
Jul	0.009 (2.6)	0.008 (1.0)	0.040 (2.3)	0.017 (3.0)	0.06 (2.4)	51 (1.4)
Aug	0.008 (2.8)	0.000 (1.0)	0.040 (2.4)	0.015 (4.2)	0.05 (2.7)	46 (1.4)
Sep	0.008 (3.0)	−0.001 (1.0)	0.039 (2.5)	0.013 (5.3)	0.05 (2.8)	43 (1.4)
Oct	0.007 (2.7)	−0.005 (1.0)	0.038 (2.3)	0.011 (3.6)	0.05 (2.5)	44 (1.6)
Nov	0.008 (2.7)	−0.001 (1.0)	0.038 (2.2)	0.011 (3.0)	0.05 (2.3)	45 (2.1)
Dec	0.009 (3.0)	0.025 (1.1)	0.040 (2.4)	0.010 (2.4)	0.05 (2.4)	48 (2.5)
Annual	0.010 (2.9)	0.027 (1.1)	0.043 (2.3)	0.013 (2.3)	0.06 (2.3)	62 (1.7)

^a^(N-depo case) – (CTL_case), ^b^(N-depo case)/(CTL_case), ^c^average at the sea surface (0–9 m), ^d^unit: µmol/L, ^e^unit: mg/m^3^, ^f^integrated over 0–200 m, ^g^unit: mg C/m^2^/day.

### Response of the surface marine ecosystem to atmospheric nitrogen deposition

**Figure 3:**
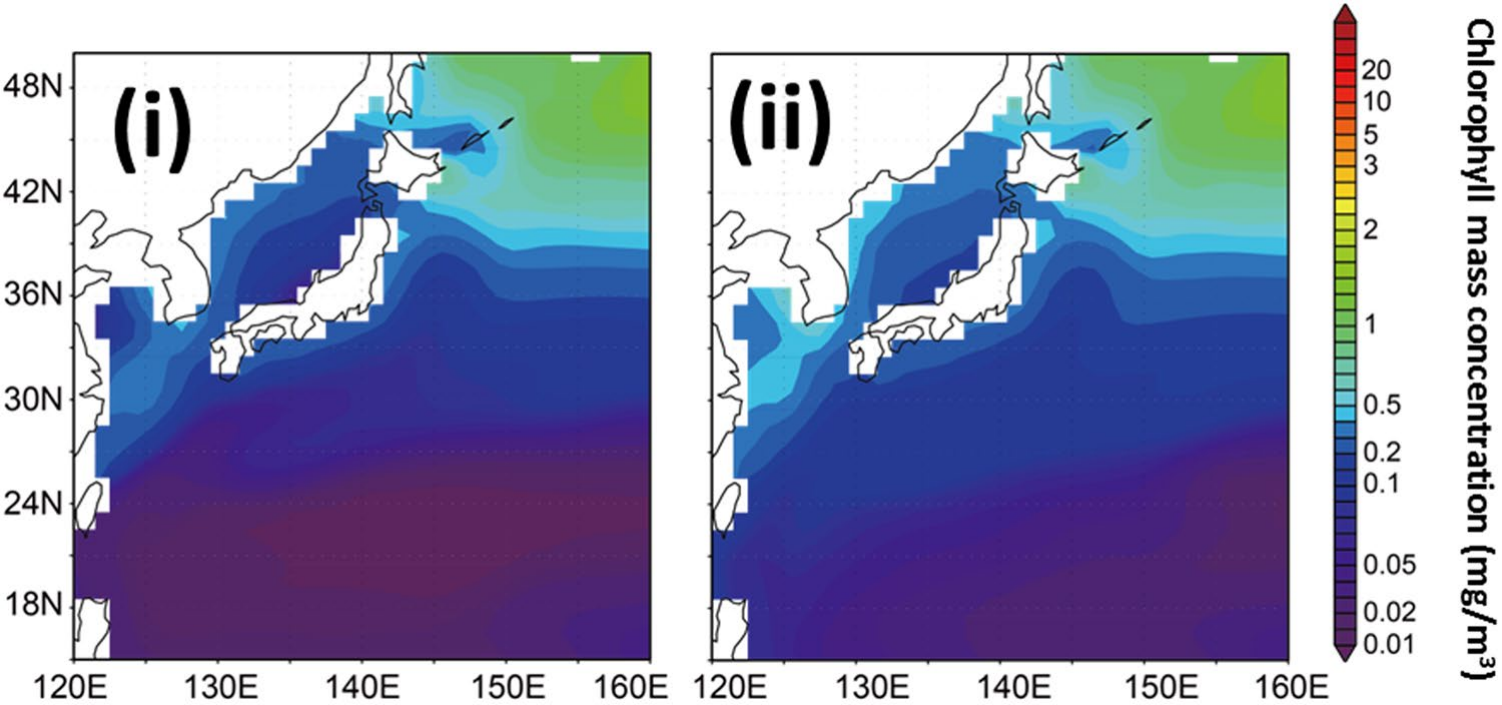
Annual mean spatial distribution of chlorophyll mass concentration at the sea surface, calculated by the COCO-NEMURO in the cases without (**i**) and with (**ii**) deposition of atmospheric inorganic nitrogen compounds. The figure is created using Grid Analysis and Display System (GrADS) Version 1.9b4 (http://cola.gmu.edu/grads/).

**Figure 4:**
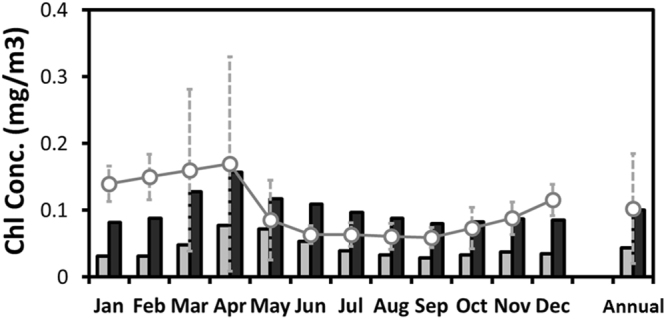
Monthly and annual means for the chlorophyll mass concentration at the sea surface for the focus area (20–30°N, 125–150°E). Gray and black bars show data in the cases without and with deposition of atmospheric inorganic nitrogen compounds, respectively. Open circles are the chlorophyll mass concentrations determined from satellite observations.

Figure 3 shows the annual mean spatial distribution of the chlorophyll mass concentration at the sea surface (averaged over 0–9 m depths) based on the CTL and N-depo cases. The spatial distributions indicate that the surface chlorophyll mass concentrations within the subtropical area increased when atmospheric nitrogen deposition was considered, whereas those in subarctic areas were almost the same. There was a sea-depth dependence in the net change in annual means of chlorophyll mass concentration between the cases within the subtropical focus area (Fig. S3). This result indicates that the change in chlorophyll mass concentration reached 150 m, with the highest contribution observed at the sea surface. Thus, atmospheric deposition can affect the chlorophyll concentration both at the sea surface and in the subsurface. Figure 4 indicates the monthly and annual means for the chlorophyll mass concentrations at the sea surface in the subtropical focus area. The annual mean chlorophyll mass concentration at the sea surface in the N-depo case was estimated to be 0.10 mg/m^3^ (compared with 0.04 mg/m^3^ in the CTL case), indicating an increase of 0.06 mg/m^3^ and corresponding to a 60% increase in the total mass concentration. This change reflects the perturbation in nutrients related to atmospheric nitrogen deposition within the marine ecosystem. The monthly mean values also increased by 1.6–2.8 times compared with the CTL case (Table 1). The effects on large and small phytoplankton, related to changes in chlorophyll mass, were also investigated (Fig. S4 and Table 1). Small phytoplankton were highly affected by atmospheric deposition and became the dominant component (70% to 81%, with an annual average of 77%), contributing most of the net change in total chlorophyll within the subtropical focus area^36^. These results suggest that atmospheric inorganic nitrogen compound depositions contribute greatly to the surface chlorophyll mass concentrations in low-nutrient subtropical areas. To compare the sea areas with sufficient nitrogen nutrients, the changes in the chlorophyll mass concentrations in a subarctic area (40–50°N, 150–160°E) were also estimated (Fig. S5). The annual mean depositions of NO_3_ and NH_4_ to this subarctic area were 19.5 and 14.9 µmol/m^2^/day, respectively, and had similar levels as the subtropical focus area did. However, the chlorophyll mass concentrations in the N-depo case were almost the same as those in the CTL case for all months, suggesting that the surface chlorophyll mass concentrations in the subarctic area were impervious to the deposition of atmospheric inorganic nitrogen compounds. This can be attributed to different factors, such as limited nutrients and radiation in the marine ecosystem.

The surface chlorophyll mass concentrations estimated from satellite observations are shown in Fig. 4 as open circles. The monthly mean sea surface chlorophyll mass concentrations for the subtropical focus area in the CTL case derived from the COCO-NEMURO were always underestimated compared with those based on satellite observations. However, the annual average of the simulated chlorophyll mass concentration reached the same level as the observational values when the deposition of atmospheric inorganic nitrogen compounds was taken into account (N-depo case). Although the surface chlorophyll mass concentrations in the N-depo case tended to be overestimated in summer and underestimated in winter, the simulated variability had a better agreement with the observations. The root-mean-square error for the monthly mean chlorophyll mass concentration in the N-depo case was approximately 50% lower than that in the CTL case (0.071 mg/m^3^). This result suggests that the nitrogen nutrient input from the atmosphere could have a significant effect on the chlorophyll mass concentrations in subtropical areas.

**Figure 5:**
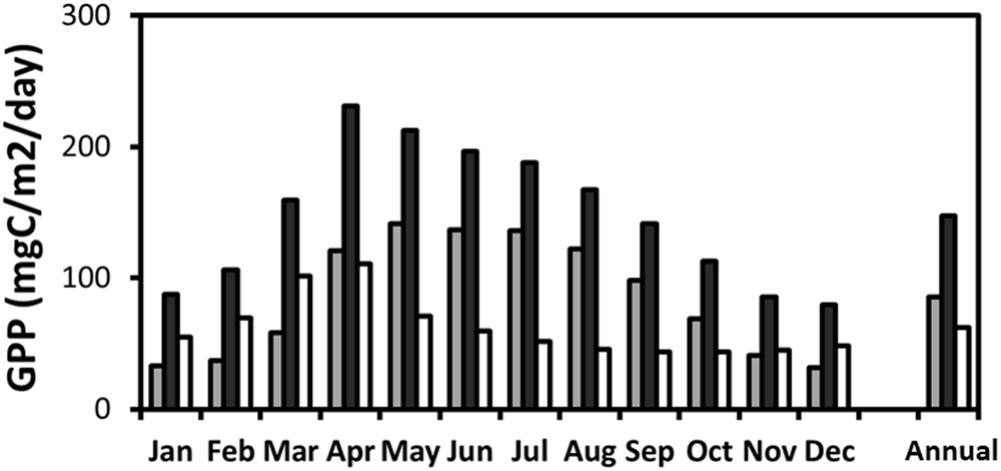
Monthly and annual means for gross primary productivities (GPPs) integrated to 200-m depth from the sea surface for the focus area (20–30°N, 125–150°E). Gray, black, and white bars indicate data for the cases without and with deposition of atmospheric inorganic nitrogen compounds and the difference between these cases, respectively.

Figure 5 shows the monthly mean results for gross primary productivity (GPP) at sea level, integrated from 0 to 200 m in depth, as calculated by COCO-NEMURO in both CTL and N-depo cases. The monthly mean GPP also increased in the range of 43–111 mg C/m^2^/day, indicating that the growth rates in the N-depo case were 1.4–2.9 times greater than those in the CTL case (Table 1). The annual mean increase was 62 mg C/m^2^/day, reflecting a 1.7-fold increase compared with the values in the CTL case. Matsumoto *et al*. reported seasonal values of GPP from ship-based observations for a site located at 30°N and 145°E, which is in the northeast of the focus area^37^. They estimated the GPP to be 396–1289 mg C/m^2^/day. The GPPs calculated by the COCO-NEMURO were much lower than these observational values, even when atmospheric nitrogen deposition was considered. This result may reflect the location of the site. However, it should be noted that the GPP value calculated using the COCO-NEMURO was improved when the atmospheric nitrogen deposition was considered.

The monthly trends in net change of chlorophyll concentration and GPP did not correspond to the atmospheric nitrogen compound deposition trends. The GPP had highest values in spring, while the highest deposition amounts occurred in winter (Fig. 2). This occurs because the phytoplankton growth in marine ecosystems is largely controlled not only by the amount of nutrients but also by the conditions of stratification, temperature and radiation in the ocean^38^.

Although we have shown the potential effects of the deposition of the atmospheric inorganic nitrogen compounds to the marine ecosystem, there are still several processes that we have not yet considered. Effects related to nitrogen (N_2_) fixation^39–42^, deposition of organic nitrogen compounds^22,24^, deposition of other nutrients (such as Fe and P from dust and/or anthropogenic activity), and the mesoscale eddy-driven upwelling^33^ are not negligible in subtropical regions. In addition, nutrients derived from river discharge^24^ may not be negligible in marginal sea areas. For example, the organic nitrogen compounds were estimated to be approximately 30% of the total nitrogen compounds in the aerosol at North Western Pacific Ocean^22^, suggesting that more nutrient deposition would be expected if they were bioavailable. The N_2_ fixation estimate is also reported for the western and central North Pacific^41,42^. The estimates for the oligotrophic North Pacific were in the range of approximately 1–100 μmol N/m^2^/day, suggesting a comparable contribution from the atmospheric nitrogen deposition. Thus, marine ecosystems have even more potential nutrient sources. Such sources should be considered in future modelling. Finally, the lack of *in situ* observational marine and atmospheric data for the subtropical focus area makes the verification of our model difficult. Clearly, more observations for subtropical areas of the North Western Pacific Ocean are needed.

We estimated the contribution of the deposition of the atmospheric inorganic nitrogen compounds to the marine ecosystem using a coupled model. An emission sensitivity analysis indicated that the sources for these depositing inorganic nitrogen compounds in the subtropical area in the North Western Pacific Ocean were mainly from East Asia. The results suggest that anthropogenic emissions from East Asia have the potential to influence these marine ecosystems. Our modelling showed that the deposition of atmospheric inorganic nitrogen compounds led to large increases in both the annual mean surface chlorophyll concentrations and GPPs, increasing their values by factors of 2.3 and 1.7 compared with cases in which deposition in subtropical regions of the North Western Pacific Ocean is not considered. In contrast, such deposition does not have a large influence in subarctic regions, indicating that the contribution of atmospheric nitrogen deposition is dependent on sea area in the North Western Pacific Ocean. A high potential for deposition of atmospheric inorganic nitrogen produced in East Asia into the marine ecosystem in the subtropical region was indicated in this study.

## Methods

### Regional atmospheric chemical transport model

To estimate the dry and wet depositions of inorganic nitrogen compounds from the East Asian air-mass into the North Western Pacific Ocean, the Community Multi-scale Air Quality (CMAQ) model^43^ (version 4.7.1) coupled with the Weather Research and Forecasting (WRF) model^44^ (version 3.3.1) was employed in this study. The configuration of the WRF-CMAQ model in this study was identical to that used in our previous work^34,35,45^. Therefore, only important details relevant to this study are given here.

The model domain, centered at 30°N and 115°E on a Lambert conformal projection, consisted of 97 × 77 horizontal grids with a resolution of 80 km. This covered the entire East Asian region (Fig. 1 in Ikeda *et al*.^35^). It also had 37 levels in the vertical direction. The simulation in this study was performed for 2009–2016, with a 1-month spin-up calculation in December 2008. The initial and boundary conditions for the WRF simulation were obtained from the National Center for Environmental Prediction Final Operational Global Analysis (FNL, ds083.2) data (six-hourly; 1° × 1° resolution). Monthly anthropogenic emissions were obtained from the Regional Emission inventory in Asia (REAS; version 2), with a 0.25° × 0.25° resolution^46^. The REAS inventory includes gases and aerosols from anthropogenic sources, such as fuel combustion, industrial processes, and agricultural activities. The satellite analysis^47,48^ indicated that the observed NO_2_ in China increased from 2005 to 2011 and then decreased to 2015. The NO_2_ levels in 2015 were almost the same as or slightly higher than those in 2008. In this study, the REAS emissions for the latest available year (2008) were employed as a representative emission level for 2009–2016 in the CMAQ simulations. Biomass burning emissions were taken from the Global Fire Emission Database (GFED; version 3.1) for the year 2010. Biogenic emission data were taken from the Model of Emissions of Gases and Aerosols from Nature (MEGAN; version 2)^49^ for the year 2000. Volcanic emission data for SO_2_ were based on Streets *et al*.^50^ for the year 2000. However, because the Miyakejima volcano was erupting during the study period, its emission was modified to 1000 tons/day based on observations made by the Japan Meteorological Agency in 2010. In addition, the AERO5 (a fifth-generation CMAQ aerosol module), with three modes to treat aerosol size distribution, and the ISORROPIA inorganic thermodynamic equilibrium module were used in our model^51,52^.

To estimate the contribution of source type (anthropogenic or natural emissions) and regions (China, Korea, and Japan within East Asia) to the deposition of inorganic nitrogen compounds, we performed a set of sensitivity simulations for 2010, in which anthropogenic emissions from China, Korea, and Japan and the natural emissions were perturbed by 20%. Details are described in our previous work^34,35^ and in the Supporting Information (TextS1).

### Three-dimensional lower trophic-marine ecosystem model

To estimate the effects of atmospheric inorganic nitrogen input as nutrients on the marine ecosystem, the North Pacific Ecosystem Model for Understanding Regional Oceanography (NEMURO)^53,54^ and Center for Climate System Research (CCSR) Ocean Component Model (COCO; version 4.9)^55,56^ were employed to evaluate biological and physical aspects, respectively. The configuration of the joint model, COCO-NEMURO, was the same as that in our previous work^53,54^. Additionally, the model performance was validated^53,57,58^. Therefore, only important details pertaining to the current study are given here.

The NEMURO consists of 11 compartments with two categories of phytoplankton: small phytoplankton (PS; such as coccolithophorids and flagellates) and large phytoplankton (PL; diatoms). The model also includes three categories of zooplankton: small zooplankton (ZS; such as foraminifera and zooflagellates), large zooplankton (ZL; copepods), and predatory zooplankton (ZP; all predators of other plankton such as Euphausiacea (krill) and jellyfish). Nitrate (NO_3_), ammonium (NH_4_), silicic acid (Si(OH)_4_), particulate organic nitrogen (PON), and dissolved organic nitrogen (DON) are also included. The chlorophyll-a (Chl-a) mass concentration and primary productivity are calculated using a C/Chl-a ratio of 60^59^ and the Redfield ratio (C/N = 106/16)^60^. The horizontal resolution of this model is 1° × 0.5–1°; it has 63 vertical layers. Sea surface forcing was derived from monthly mean surface flux climatology compiled by Röske^61^. The observed nitrate and silicate concentrations used as the initial conditions were obtained from the World Ocean Atlas 2013 dataset^62^.

### Coupling of atmospheric and marine ecosystem models

We used the dry and wet deposition amounts for inorganic nitrogen, given as fine- (summed in Aitken and accumulation modes) and coarse-mode aerosol particles, and for gases as input into the NEMURO. A monthly mean dataset was collated for dry and wet deposition for inorganic nitrogen compounds for each grid of the North Western Pacific Ocean in the model domain, based on averages for 2009–2016. These values were input into the NEMURO because the COCO-NEMURO does not have a process to input atmospheric nutrients. Therefore, we first needed to establish a pathway for a new nitrogen nutrient supply from the atmosphere. In this study, we assumed that the atmospheric inorganic nitrogen compounds (NO_3_^−^, HNO_3_, NH_4_^+^, and NH_3_) that were calculated in the WRF-CMAQ model as being deposited on the sea surface were all bioavailable nitrogen sources for small and large phytoplankton in the COCO-NEMURO. The WRF-CMAQ model results were assigned to atmospheric nitrate [NO_3_ = NO_3_^−^ (particles) +HNO_3_ (gas)] or ammonium [NH_4_ = NH_4_^+^ (particles) +NH_3_ (gas)] deposition. These amounts were added as nitrate or ammonium compartments in the COCO-NEMURO for the sea surface layer, which is covered by the WRF-CMAQ model’s domain. To incorporate the deposition data for both NO_3_ and NH_4_, as calculated by the WRF-CMAQ model, into the COCO-NEMURO, the data were re-gridded to the COCO-NEMURO grid cells via area-weighting.

The physical model of the COCO was run without the NEMURO for 3000 simulated years, with the final state regarded as being at equilibrium. Once this state was reached, the COCO was coupled with the NEMURO and spun up for 120 years for simulations with and without monthly mean values of atmospheric inorganic nitrogen deposition for (NH_4_ and NO_3_) generated by the WRF-CMAQ model’s dataset. The results for the average of the last 20 years with and without nitrogen deposition were used for discussion.

### Satellite data analysis

To compare the surface chlorophyll concentrations estimated by the COCO-NEMURO, Aqua-MODIS (Moderate Resolution Imaging Spectroradiometer) Level-3 chlorophyll-a concentration data from January 2009 to December 2016 were used, obtained from NASA GSFC’s Ocean Color website (http://oceancolor.gsfc.nasa.gov/). We used monthly composite chlorophyll-a data with a 9-km spatial resolution retrieved for the focus areas.

### Data Availability

The modelling data are available from the authors upon request.

## Electronic supplementary material

Supporting Information

## References

[1] Baker AR (2017). Observation- and model-based estimates of particulate dry nitrogen deposition to the oceans. Atmos. Chem. Phys..

[2] Duce RA (2008). Impacts of atmospheric anthropogenic nitrogen on the open ocean. Science.

[3] Guieu C (2014). The significance of the episodic nature of atmospheric deposition to Low Nutrient Low Chlorophyll regions. Global Biogeochem. Cy..

[4] Krishnamurthy A, Moore JK, Zender CS, Luo C (2007). Effects of atmospheric inorganic nitrogen deposition on ocean biogeochemistry. J. Geophys. Res..

[5] Jickells TD (2005). Global iron connections between desert dust, ocean biogeochemistry, and climate. Science.

[6] Jickells TD (2017). A reevaluation of the magnitude and impacts of anthropogenic atmospheric nitrogen inputs on the ocean. Global Biogeochem. Cy..

[7] Ito A (2015). Atmospheric processing of combustion aerosols as a source of bioavailable iron. Environ. Sci. Technol. Lett..

[8] Letscher RT, François Primeau J, Moore K (2016). Nutrient budgets in the subtropical ocean gyres dominated by lateral transport. Nat. Geosci..

[9] Onitsuka G, Uno I, Yanagi T, Yoon J-H (2009). Modeling the Effects of Atmospheric Nitrogen Input on Biological Production in the Japan Sea. J Oceanogr..

[10] Richon, C. *et al*. Modeling the impacts of atmospheric deposition of nitrogen and desert dust-derived phosphorus on nutrients and biological budgets of the Mediterranean Sea in *Progress in Oceanography*, 10.1016/j.pocean.2017.04.009 (2017).

[11] Suntharalingam P (2012). Quantifying the impact of anthropogenic nitrogen deposition on oceanic nitrous oxide. Geophys. Res. Lett..

[12] Yang S, Gruber N (2016). The anthropogenic perturbation of the marine nitrogen cycle by atmospheric deposition: Nitrogen cycle feedbacks and the ^15^N Haber-Bosch effect. Global Biogeochem. Cy..

[13] Ohara T (2007). Asian emission inventory for anthropogenic emission sources during the period 1980–2020. Atmos. Chem. Phys..

[14] Itahashi S, Uno I, Irie H, Kurokawa J-I, Ohara T (2014). Regional modeling of tropospheric NO_2_ vertical column density over East Asia during the period 2000–2010: comparison with multisatellite observations. Atmos. Chem. Phys..

[15] Furutani H, Meguro A, Iguchi H, Uematsu M (2010). Geographical distribution and sources of phosphorus in atmospheric aerosol over the North Pacific Ocean. Geophys. Res. Lett..

[16] Geng H, Park Y, Hwang H, Kang S, Ro C-U (2009). Elevated nitrogen-containing particles observed in Asian dust aerosol samples collected at the marine boundary layer of the Bohai Sea and the Yellow Sea. Atmos. Chem. Phys..

[17] Iwamoto Y, Uematsu M (2014). Spatial variation of biogenic and crustal elements in suspended particulate matter from surface waters of the North Pacific and its marginal seas. Prog. Oceanogr..

[18] Jung J, Furutani H, Uematsu M (2011). Atmospheric inorganic nitrogen in marine aerosol and precipitation and its deposition to the North and South Pacific Oceans. J. Atmos. Chem..

[19] Jung J, Furutani H, Uematsu M, Kim S, Yoon S (2013). Atmospheric inorganic nitrogen input via dry, wet, and sea fog deposition to the subarctic western North Pacific Ocean. Atmos. Chem. Phys..

[20] Kim T-W, Lee K, Najjar RG, Jeong H-D, Jeong HJ (2011). Increasing N abundance in the Northwestern Pacific Ocean due to atmospheric nitrogen deposition. Science.

[21] Martino M (2014). Western Pacific atmospheric nutrient deposition fluxes, their impact on surface ocean productivity. Global Biogeochem. Cy..

[22] Miyazaki Y, Kawamura K, Jung J, Furutani H, Uematsu M (2011). Latitudinal distributions of organic nitrogen and organic carbon in marine aerosols over the western North Pacific. Atmos. Chem. Phys..

[23] Nakamura T, Matsumoto KI, Uematsu M (2005). Chemical characteristics of aerosols transported from Asia to the East China Sea: an evaluation of anthropogenic combined nitrogen deposition in autumn. Atmos. Environ..

[24] Nakamura T, Ogawa H, Maripi DK, Uematsu M (2006). Contribution of water soluble organic nitrogen to total nitrogen in marine aerosols over the East China Sea and western North Pacific. Atmos. Environ..

[25] Uematsu M (2010). (2010) Atmospheric transport and deposition of anthropogenic substances from the Asia to the East China Sea. Mar. Chem..

[26] Dentener F (2006). Nitrogen and sulfur deposition on regional and global scales: A multimodel evaluation. Global Biogeochem. Cy..

[27] Itahashi S, Hayami H, Uno I, Pan X, Uematsu M (2016). Importance of coarse-mode nitrate produced via sea salt as atmospheric input to East Asian oceans. Geophys. Res. Lett..

[28] Uno I (2007). Numerical study of the atmospheric input of anthropogenic total nitrate to the marginal seas in the western North Pacific region. Geophys. Res. Lett..

[29] Zhang Y, Yu Q, Ma W, Chen L (2010). Atmospheric deposition of inorganic nitrogen to the eastern China seas and its implications to marine biogeochemistry. J. Geophys. Res..

[30] Onitsuka G, Yanagi T, Yoon J-H (2007). A numerical study on nutrient sources in the surface layer of the Japan Sea using a coupled physical-ecosystem model. J. Geophys. Res..

[31] Mulholland, M. & Lomas, M. Nitrogen uptake and assimilation in Nitrogen in the marine environment (eds Capone, D. G., Bronk, D. A., Mulholland, M. R. & Carpenter, E. J.) 303–384 (Elsevier, 2008).

[32] Shiomoto A, Sasaki K, Shimoda T, Matsumura S (1994). Kinetics of nitrate and ammonium uptake by the natural populations of marine phytoplankton in the surface water of the Oyashio region during spring and summer. J. Oceanogr..

[33] Honda, M. C., Sasai, Y., Siswanto, E. Kuwano-Yoshida, A. & Cronin M. F. Impact of cyclonic eddies on biogeochemistry in the oligotrophic ocean based on biogeochemical/physical/meteorological time-series at station KEO submitted to *Prog Earth Planet Sci*. (2017)

[34] Ikeda K (2014). Sensitivity analysis of source regions to PM2.5 concentrations at Fukue Island, Japan. J. Air Waste Manag. Assoc..

[35] Ikeda K (2015). Source region attribution of PM2.5 mass concentrations over Japan. Geochem. J..

[36] Irwin AJ, Finkel ZV, Schofield OME, Falkowski PG (2006). Scaling-up from nutrient physiology to the size-structure of phytoplankton communities. J. Plank. Res..

[37] Matsumoto K, Abe O, Fujiki T, Sukigara C, Mino Y (2016). Primary productivity at the time-series stations in the northwestern Pacific Ocean: is the subtropical station unproductive?. J. Oceanogr..

[38] Sverdrup H (1953). On conditions for the vernal blooming of phytoplankton. J. Cons. Perm. Int. Explor. Mer..

[39] Karl DM (1997). The role of nitrogen fixation in biogeochemical cycling in the subtropical North pacific Ocean. Nature.

[40] Kim D (2017). The reduction in the biomass of cyanobacterial N2 fixer and the biological pump in the Northwestern Pacific Ocean. Sci. Rep..

[41] Shiozaki T (2010). New estimation of N2 fixation in the western and central Pacific Ocean and its marginal seas. Global Biogeochem. Cycles.

[42] Luo Y-W (2014). Data-based assessment of environmental controls on global marine nitrogen fixation. Biogeosciences.

[43] Byun D, Schere KL (2006). Review of the governing equations, computational algorithms, and other components of the Models-3 Community Multiscale Air Quality (CMAQ) modeling system overview. Appl. Mech. Rev..

[44] Skamarock, W. C. *et al*. A description of the Advanced Research WRF Version 3. NCAR Technical Note NCAR/TN-475+ STR (NCAR, 2008).

[45] Yamaji K, Ikeda K, Irie H, Kurokawa J, Ohara T (2014). Influence of model grid resolution on NO_2_ vertical column densities over East Asia. J. Air Waste Manage. Assoc..

[46] Kurokawa J (2013). Emissions of air pollutants and green-house gases over Asian regions during 2000–2008: Regional Emission inventory in Asia (REAS) version 2. Atmos. Chem. Phys..

[47] Irie H (2016). Turnaround of tropospheric nitrogen dioxide pollution trends in China, Japan, and South Korea. SOLA.

[48] Liu L (2017). Temporal characteristics of atmospheric ammonia and nitrogen dioxide over China based on emission data, satellite observations and atmospheric transport modeling since 1980. Atmos. Chem. Phys..

[49] Guenther A (2006). Estimates of global terrestrial isoprene emissions using MEGAN (Model of Emissions of Gases and Aerosols from Nature). Atmos. Chem. Phys..

[50] Streets DG (2003). An inventory of gaseous and primary aerosol emissions in Asia in the year 2000. J. Geophys. Res..

[51] Nenes A, Pilinis C, Pandis SN (1998). ISORROPIA: a new thermodynamic equilibrium model for multiphase multicomponent marine aerosols. Aquat. Geochem..

[52] Nenes A, Pilinis C, Pandis SN (1999). Continued development and testing of a new thermodynamic aerosol module for urban and regional air quality models. Atmos. Environ..

[53] Aita, M. N., Yamanaka, Y. & Kishi, M. J. Interdecadal variation of the lower trophic ecosystem in the Northern Pacific between 1948 and 2002, in a 3-D implementation of the NEMURO model. *Ecol*. *Modelll*, 10.1016/j.ecolmodel.2006.07.045 (2007).

[54] Kishi, M. J. *et al*. NEMURO – Introduction to a lower trophic level model for the North Pacific marine ecosystem model. *Ecol*. *Modell*, 10.1016/j.ecolmodel.2006.08.021 (2007).

[55] Hasumi, H. CCST Ocean Component Model (COCO) version 4.0 (Rep. Cent. Clim. Syst. Res., 2006).

[56] Tatebe H, Hasumi H (2010). Formation mechanism of the Pacific equatorial thermocline revealed by a general circulation model with a high accuracy tracer advection scheme. Ocean Model..

[57] Yoshie, N, *et a*l. Parameter sensitivity study of the NEMURO lower trophic level marine ecosystem model. *Ecol*. *Modell*, 10.1016/j.ecolmodel.2006.07.043 (2007)

[58] Fujii, M. *et al*. Comparison of seasonal characteristics in biogeochemistry among the subarctic North Pacific stations described with a NEMURO-based marine ecosystem model. *Ecol*. *Modell*, 10.1016/j.ecolmodel.2006.02.046 (2007)

[59] Fasham MJR, Ducklow HW, McKelvie SM (1990). A nitrogen-based model of plankton dynamics in the oceanic mixed layer. J. Mar. Res..

[60] Redfield, A. C., Ketchum, B. H. & Richards, F. A. The influence of organisms on the composition of seawater in The Sea, Volume 2 (ed. Hill, M. N.) 26–77 (Wiley-Interscience, 1963).

[61] Röske F (2005). Global oceanic heat and fresh water forcing datasets based on ERA-40 and ERA-15 Max-Planck-Institut fuerMeteorologie. . Reports on Earth System Science.

[62] Garcia HE (2013). World Ocean Atlas 2013: Volume 4: Dissolved InorganicNutrients (phosphate, nitrate, silicate). Technical Ed. NOAA Atlas NESDIS.

