# Supplementary material for: Seasonal Response of North Western Pacific Marine Ecosystems to Deposition of Atmospheric Inorganic Nitrogen Compounds from East Asia
Source: Sci Rep. 2018 Jun 29;8:9324. doi: 10.1038/s41598-018-27523-w (PMC6026176; doi:10.1038/s41598-018-27523-w)
Supplement: Supplementary file 1 — Supporting Information [file 41598_2018_27523_MOESM1_ESM.docx]

# Supporting Information for “Seasonal Response of North Western Pacific Marine Ecosystems to Deposition of Atmospheric Inorganic Nitrogen Compounds from East Asia”

Fumikazu Taketani^1,*^, Maki N. Aita^1^, Kazuyo Yamaji^1,2^, Takashi Sekiya^3^, Kohei Ikeda^1,4^, Kosei Sasaoka^1^, Taketo Hashioka^1^, Makio C. Honda^1^, Kazuhiko Matsumoto^1^, and Yugo Kanaya^1^

^1^Research and Development Center for Global Change, Japan Agency for Marine-Earth Science and Technology, 3173-25 Showa-machi, Kanazawa-ku, Yokohama, Kanagawa 236-0001, Japan

^2^Graduate School of Maritime Sciences, Kobe University, 5-1-5 Fukae-minamimachi, Higashinada-ku, Kobe, Hyogo 658-0022, Japan

^3^Project Team for HPC Advanced Predictions utilizing Big Data, Japan Agency for Marine-Earth Science and Technology, 3173-25 Showa-machi, Kanazawa-ku, Yokohama, Kanagawa 236-0001, Japan

^4^Center for Global Environmental Research, National Institute for Environmental Studies, 16-2 Onogawa, Tsukuba, Ibaraki 305-8506, Japan

## Text S1

*Analysis of source contributions*

We performed sensitivity simulations for 2010. The NO_2_ level averaged for 2009 -2015 by satellite analysis^1,2^ was almost same as the level in 2010. Therefore, we used the data for 2010 as the typical case in this analysis. An emission sensitivity approach was used to estimate the contribution from each source region and source type in East Asia to the atmospheric deposition of inorganic nitrogen compounds in the subtropical focus area. This estimate was conducted using two kinds of simulations: a base run and a sensitivity run with emission perturbations. The contribution from each source region was calculated by taking the difference between the simulated depositions from the base and each sensitivity experiment. In this study, to avoid a strong nonlinear effect and ensure a detectable response, we applied a 20% reduction in anthropogenic emissions from each source region (China, Korea, and Japan) in our sensitivity simulations^3,4^. The contribution of natural sources was quantified using sensitivity simulations, in which the emissions from biomass burning, biogenic sources, and volcanoes were simultaneously perturbed within the model domain.

The contribution from a source *s* to the deposition amount in a focus area, resulting from the −20% sensitivity simulations, *A_s_*_, -20%_, was estimated as follows:

$A_{s,-20\%}=5\times(C_{\text{base}}-C_{s,-20\%}),$ (1)

where *C*_base_ is the concentration derived from the base run in which all of the emission sources were included and *C_s_*_, -20%_ is the deposition derived from the sensitivity simulation with emissions from source *s* perturbed by −20%. The absolute contribution from each source was obtained by multiplying the difference in simulated deposition between the base and sensitivity runs by five, assuming a linear relationship. The ratios of various contributions to the total were defined as the proportion of the contribution from each source *s* to the total value of contributions from all emission sources:

$R_{s} (\%) =\frac{A_{s,-20\%}}{C_{base}}\times100.$ (2)

## References

1. Liu, L. et al. Temporal characteristics of atmospheric ammonia and nitrogen dioxide over China based on emission data, satellite observations and atmospheric transport modeling since 1980, *Atmos. Chem. Phys.*, **17**, 9365-9378, https://doi.org/10.5194/acp-17-9365-2017, (2017).

2. Irie, et al. Turnaround of tropospheric nitrogen dioxide pollution trends in China, Japan, and South Korea, *SOLA*, **12**, 170–174, (2016).

3. Ikeda, K. *et al.* Source region attribution of PM2.5 mass concentrations over Japan. *Geochem. J.*, **49,** 185–194 (2015).

4. Ikeda, K. et al*.* Sensitivity analysis of source regions to PM2.5 concentrations at Fukue Island, Japan. *J. Air Waste Manag. Assoc.*, **64,** 445–452 (2014).

## Supplementary tables

**Table S1** Monthly and annual mean deposition fluxes and their breakdown into processes and chemical forms of NH_4_ for the subtropical focus area

| Month | Total  deposition^a^ |  | Dry deposition | | |  | Wet deposition | | |
| --- | --- | --- | --- | --- | --- | --- | --- | --- | --- |
|  |  |  | Gas | Fine  particles | Coarse  particles |  | Gas | Fine  particles | Coarse  particles |
| Jan | 38.4 |  | 11.0% | 3.9% | 9.8% |  | -^b^ | 71.6% | 3.7% |
| Feb | 33.9 |  | 10.1% | 3.9% | 8.0% |  | -^b^ | 74.6% | 3.4% |
| Mar | 26.5 |  | 9.5% | 4.1% | 8.0% |  | -^b^ | 75.3% | 3.1% |
| Apr | 26.8 |  | 5.7% | 2.7% | 3.3% |  | -^b^ | 85.8% | 2.5% |
| May | 17.8 |  | 2.9% | 2.7% | 1.2% |  | -^b^ | 91.6% | 1.6% |
| Jun | 11.4 |  | 2.4% | 1.7% | 0.8% |  | -^b^ | 93.4% | 1.7% |
| Jul | 7.9 |  | 3.6% | 1.8% | 0.9% |  | -^b^ | 91.9% | 1.8% |
| Aug | 7.5 |  | 4.1% | 2.7% | 1.1% |  | -^b^ | 90.1% | 2.0% |
| Sep | 8.9 |  | 3.3% | 3.4% | 1.4% |  | -^b^ | 90.0% | 1.8% |
| Oct | 10.3 |  | 8.5% | 4.9% | 4.1% |  | -^b^ | 80.0% | 2.6% |
| Nov | 15.5 |  | 11.1% | 4.4% | 7.9% |  | -^b^ | 74.0% | 2.6% |
| Dec | 30.6 |  | 13.5% | 3.7% | 10.7% |  | -^b^ | 68.1% | 3.9% |
| Annual | 19.6 |  | 8.5% | 3.5% | 6.4% |  | -^b^ | 78.7% | 2.9% |

^a^Unit: µmol/m^2^/day, total deposition: NH_4_ (total)= NH_4_^+^ (particles) + NH_3_ (gas); ^b^value below 0.1%

**Table S2** Monthly and annual mean deposition fluxes and their breakdown to processes and chemical forms of NO_3_ for the subtropical focus area

| Month | Total deposition^a^ |  | Dry deposition | | |  | Wet deposition | | |
| --- | --- | --- | --- | --- | --- | --- | --- | --- | --- |
|  |  |  | Gas | Fine  particles | Coarse  particles |  | Gas | Fine  particles | Coarse  particles |
| Jan | 57.5 |  | 13.6% | 0.5% | 44.0% |  | -^b^ | 21.0% | 20.9% |
| Feb | 46.3 |  | 14.5% | 0.4% | 43.4% |  | -^b^ | 19.9% | 21.8% |
| Mar | 37.0 |  | 15.7% | 0.3% | 47.5% |  | -^b^ | 15.0% | 21.6% |
| Apr | 30.6 |  | 13.7% | 0.1% | 35.5% |  | -^b^ | 21.6% | 29.1% |
| May | 24.1 |  | 15.5% | -^b^ | 23.1% |  | -^b^ | 32.1% | 29.2% |
| Jun | 16.1 |  | 10.7% | -^b^ | 15.7% |  | -^b^ | 42.4% | 31.2% |
| Jul | 14.1 |  | 7.7% | 0.1% | 13.8% |  | -^b^ | 47.8% | 30.6% |
| Aug | 14.9 |  | 9.4% | 0.2% | 15.4% |  | -^b^ | 45.1% | 29.9% |
| Sep | 16.7 |  | 10.8% | 0.1% | 23.5% |  | -^b^ | 36.6% | 29.0% |
| Oct | 20.8 |  | 11.8% | 0.2% | 38.5% |  | -^b^ | 23.0% | 26.6% |
| Nov | 31.5 |  | 13.8% | 0.1% | 44.3% |  | -^b^ | 18.0% | 23.7% |
| Dec | 50.1 |  | 14.0% | 0.4% | 44.6% |  | -^b^ | 19.0% | 22.1% |
| Annual | 30.0 |  | 13.4% | 0.3% | 37.4% |  | -^b^ | 24.3% | 24.7% |

^a^Unit: µmol/m^2^/day, total deposition: NO_3_ (total) = NO_3_^-^ (particles) + HNO_3_ (gas); ^b^value below 0.1%

## Supplementary figures

**
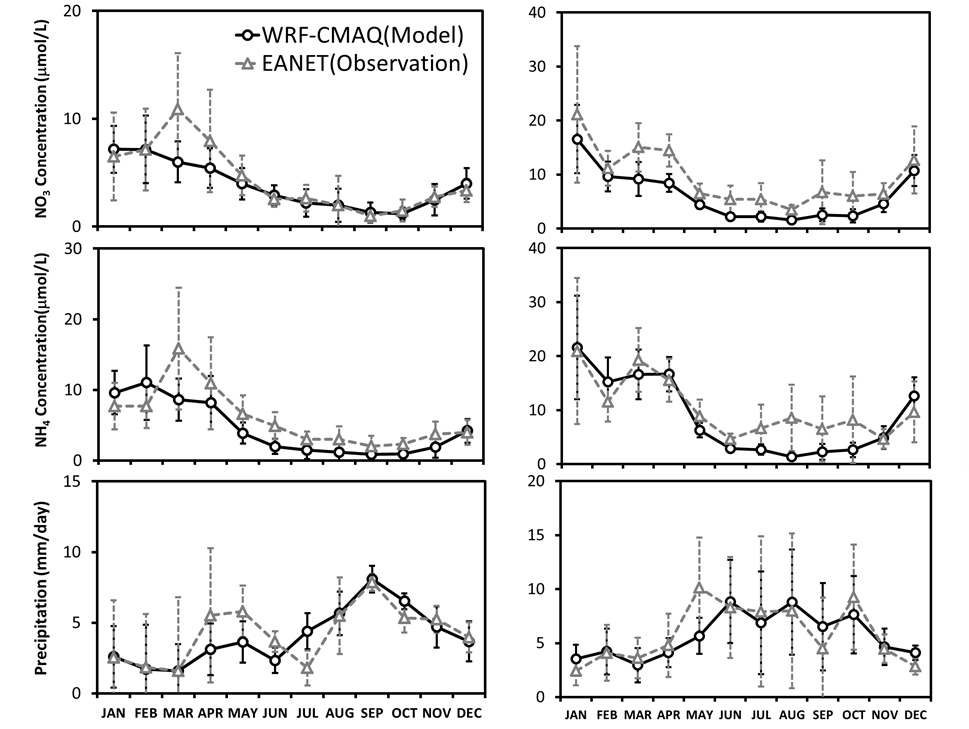
**

**Figure S1**

Monthly means of NO_3_^-^ (upper panel) and NH_4_^+^ (middle panel) concentrations in rain and other forms of precipitation (bottom panel), averaged for 2009–2015 at the two Acid Deposition Monitoring Network in East Asia (EANET) sites. Data for Ogasawara (27.05°N, 142.13°E) are shown in the left panels, and Hedo (26.52°N, 128.15°E) data are shown in the right panels. Black open circles and gray triangles indicate WRF-CMAQ model results and EANET observations, respectively.


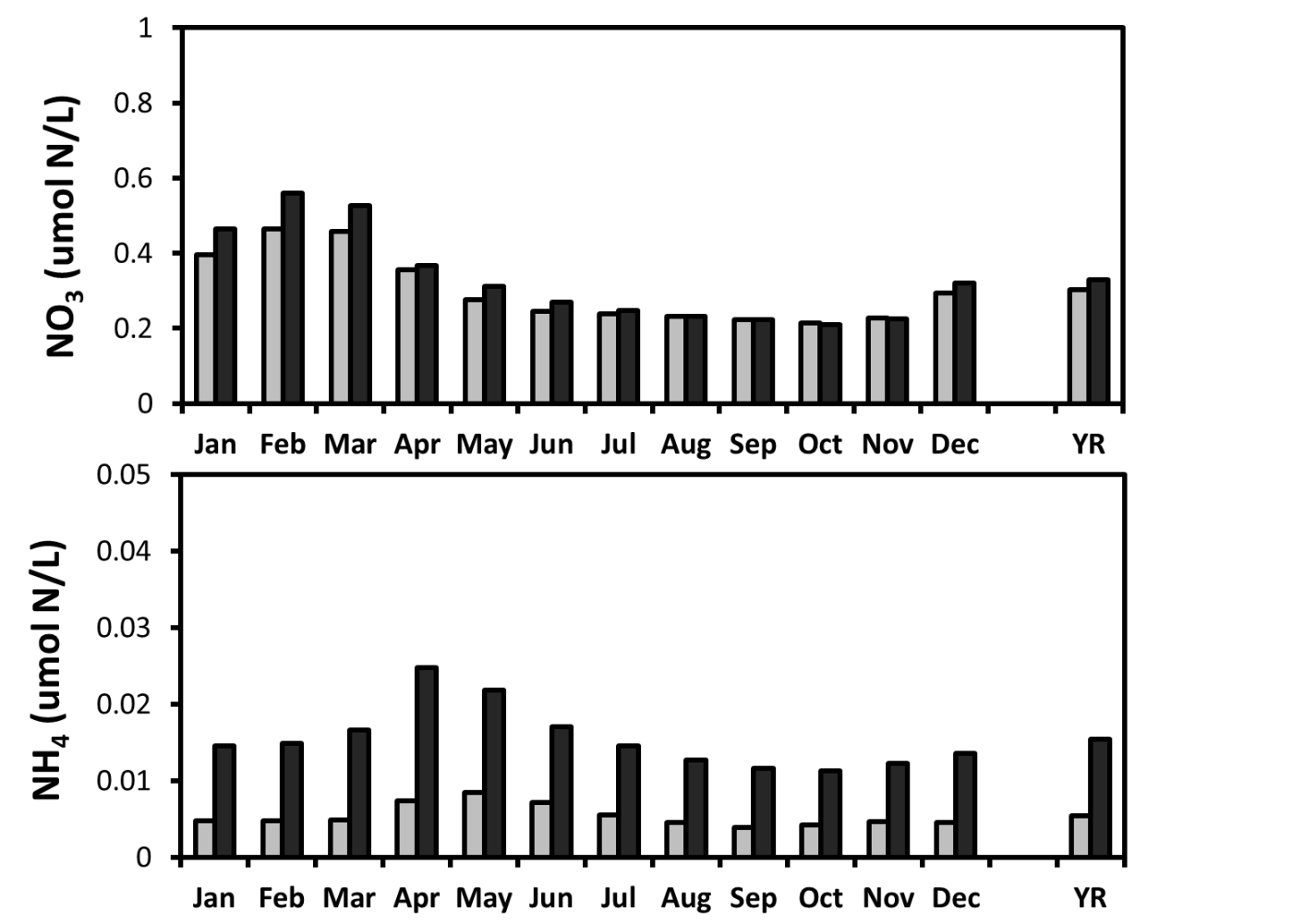


**Figure S2**

Monthly and annual means of NO_3_ and NH_4_ concentrations for the focus area (20–30°N, 125–150°E) at the sea surface. Gray and black bars show results for cases without and with deposition of atmospheric inorganic nitrogen compounds, respectively.

**
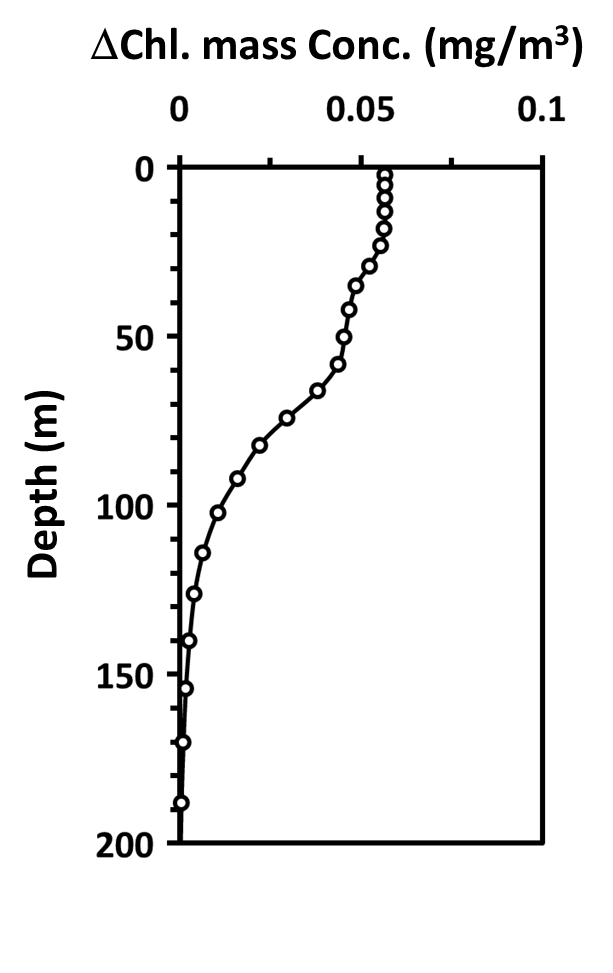
**

**Figure S3**

Annual mean values showing the sea-depth dependence of the net change in chlorophyll mass concentration between cases with and without the deposition of atmospheric inorganic nitrogen compounds, integrated from the sea surface to a 200 m depth for the subtropical focus area.


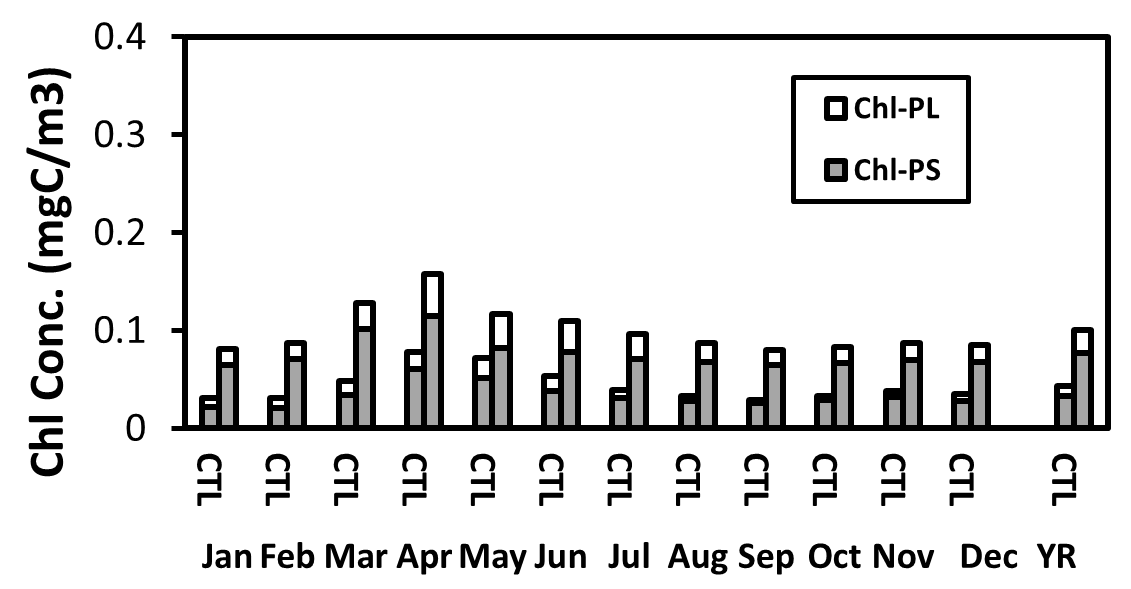


**Figure S4**

Monthly and annual mean chlorophyll mass concentrations for the focus area (20–30°N, 125–150°E) at the sea surface. Left and right side bars for each month reflect results for cases without and with deposition of atmospheric inorganic nitrogen compounds, respectively. Gray and white bars indicate contributions to chlorophyll (Chl) from small (PS) and large (PL) phytoplankton, respectively.


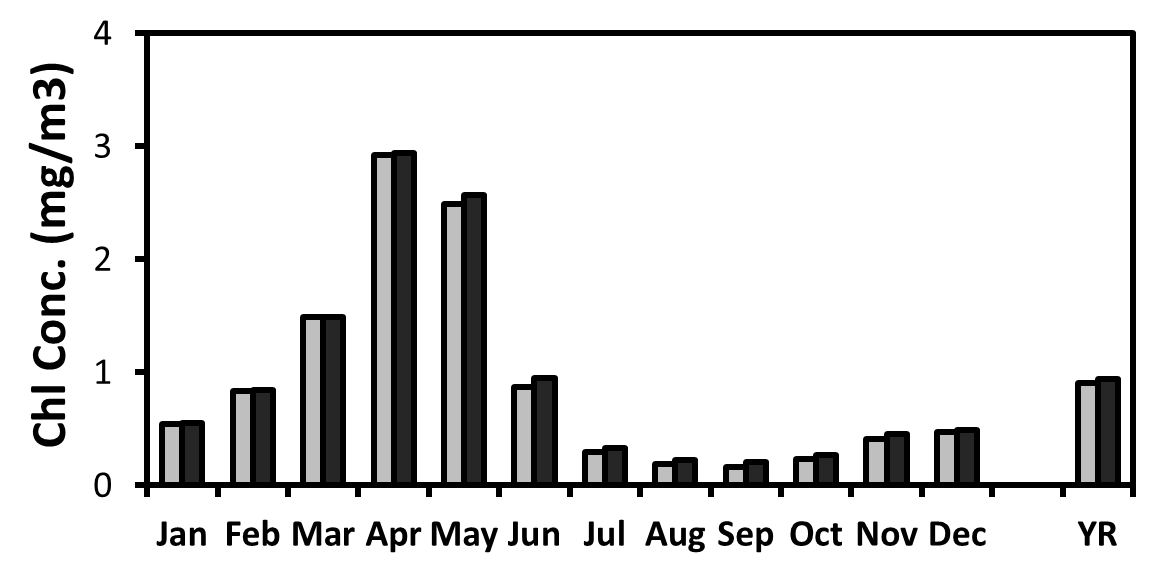


**Figure S5**

Monthly and annual mean chlorophyll mass concentrations for the subarctic focus area (40–50°N, 150–160°E) at the sea surface. Gray and black bars indicate cases without and with deposition of atmospheric inorganic nitrogen compounds, respectively.
